# Effects of a Chipboard Structure on Its Physical and Mechanical Properties

**DOI:** 10.3390/ma12223777

**Published:** 2019-11-17

**Authors:** Radosław Mirski, Adam Derkowski, Dorota Dziurka, Dorota Dukarska, Rafał Czarnecki

**Affiliations:** Department of Wood Based Materials, Faculty of Wood Technology, Poznań University of Life Sciences, Wojska Polskiego 38/42, 60-627 Poznań, Poland; rmirski@up.poznan.pl (R.M.); dorota.dziurka@up.poznan.pl (D.D.); dorota.dukarska@up.poznan.pl (D.D.); rafal.czarnecki@up.poznan.pl (R.C.)

**Keywords:** chips, sawdust, sawmill processing, chips boards, mechanical properties

## Abstract

The paper evaluated the possibility of manufacturing wood-based boards from the material left over from sawmill processing of wood. The boards were made from chips created during cant preparation for cutting and sawdust generated during further sawnwood preparation. They were made as one- and three-ply boards with face layers containing industrial microchips. Mechanical properties determined for one-ply boards in a bend test were used as guidelines for manufacturing three-ply boards. The outcomes were much better when the core layer comprised a mix of chips and sawdust than the chips alone. The study also showed that for the assumed technological parameters it is possible to produce three-ply boards with properties meeting the criteria for P2 furniture boards.

## 1. Introduction

The industry of primary wood processing is the largest recipient of wood residues. However, even in highly developed and industrialized countries, the final product in the form of sawnwood placed on the market is only 50% of the processed wood. The remaining 50% constitute a so-called material loss, of which 16% account for sawdust and 22% for chips. The remaining 12% result from the loss of thickness caused by desorption changes. Sawdust is the finest material generated during sawing and its form depends on the cutting tools used. Chips come in slightly greater dimensions. Their size largely depends on the material they are obtained from. Chips are also produced from other already fragmented materials such as edgings, impaired sawnwood, waste wood, or waste generated during a pile formation. Management of these products, often including bark, poses a serious challenge [1]. Sawmills in Poland process about 20 million cubic meters of softwood per year, thus yielding ca. 4 million cubic meters of chips and ca. 3 million cubic meters of sawdust. These materials are mostly used for manufacture of particleboards and paper. Chips, as less fragmented material, offer more practical applications. They are useful in the production of fiberboards or paper, and after further shredding they are glued into particleboards. Sawdust is added to particleboards or pellet in specific proportions supplementing the core material [2,3,4]. An advantage of using wood material in production of large-size boards is the possibility to apply all kinds of easily accessible binding agents such as UF, PF, PMDI (urea-formaldehyde, phenol-formaldehyde, polymeric diphenyl methane diisocyanate) or their hybrids [5,6,7,8,9,10,11]. For all these solutions, chips need to be broken into much smaller pieces, which requires additional energy. The fragmentation is mainly aimed at obtaining chips of specific linear dimensions and thickness, as the chip geometry guarantees the required mechanical properties of the boards [12,13,14,15,16,17,18,19]. The shape and size of the chips determine the effectiveness of glue cover and the chip orientation during the mat formation. Chip thickness translates mainly into modulus of rigidity and tensile strength perpendicular to the board plane. In general, as thickness increases, modulus of rigidity drops down and modulus of elasticity grows up. Often these parameters are impacted by the length-to-thickness ratio (slenderness). The greater the slenderness, the greater the bending strength is [20,21,22,23,24]. Usually thickness of the wood waste used for manufacturing wood-based materials range from 0.2 mm to ca. 3.2 mm. Maximum thickness of the particles does not exceed 1.2 mm (0.2–0.6 PB, 0.4–1.1 OSB/OSL). Average dimensions of wood chips reach 30–50 mm in length, 25–40 mm in width, and 4–6 mm in thickness. These dimensions are several times greater than of the chips intended for structural materials or core layer of three-ply boards. Much greater thickness of the waste chips than the proper chips (intended for the core layer of P2 furniture particleboards) means that this type of raw material cannot be used in the production of thin or medium thickness boards used in furniture industry as construction elements, i.e., in cabinet or upholstered furniture bodies or as partitions or doors for cabinet furniture. Some internal decoration products require boards with thickness exceeding 22 mm and physical and mechanical properties meeting the criteria for P2 boards, according to EN 622 standard [25]. Most often, these types of boards are refined and used for kitchen counters, office desks, or windowsills.

Therefore, the aim of this study was to determine the possibility of using waste chips as substitutes of chips intended for the core layer of furniture particleboards. The proposed solution may contribute to reducing the costs of board production due to the use of unprocessed material. Sorting intensity will be reduced to a minimum, and the shredding operation will be eliminated.

## 2. Materials and Methods

The study used sawmill chips obtained via fragmentation of mostly debarked wood material (*Pinus Sylvestris* L.) and sawdust generated during sawmill processing. The chips remaining on a 50 × 50 mm sieve were pre-sorted and large pieces of bark that passed through this sieve were manually removed. Sawdust was collected directly from a daily tank collecting material from the entire wood processing flow. Thus, the sawdust contained material obtained by both sawing and planing. All the wood material underwent a fractional analysis and was dried up to about 3.5% moisture content prior to pressing. The material was dried in a laboratory tumble drier at 120 °C (inlet)–110 °C (outlet). The process was carried out until the assumed humidity was reached. 

Both types of particles were glued with MUF (melamine-urea-formaldehyde) resin received from Pfleiderer Silekol Sp. z o.o. Ammonium nitrate (20%), added at 1.5% of the resin dry weight, was used as a curing agent for MUF resin. The chips and sawdust were glued separately. Gluing degree for chips was 6% and sawdust was added to chip-sawdust boards at the level of 8%.

The material prepared this way was used for manufacture of chipboards (PZ) and chip-sawdust (PT) boards with the following densities: 450, 525, 600, 675, and 750 kg/m^3^. The boards were 24 mm thick and sawdust to chip ratio was 70:30. Manually formed mat was pressed at 190 °C, for 20 s per mm of board thickness at a unit pressure of 1.8 MPa–3.2 MPa, with 0.35 MPa increments. Seasoned experimental boards were sampled to assess their properties in a 3-point bending test including assessment of modulus of elasticity and bending strength as described in EN 310 standard [26]. 

Industrial boards 28 mm and 38 mm thick were used as reference boards. They were assessed both intact and after removal of the face layers. Constant thickness of the face layers was assumed, as board thickness changes usually by changing the bulk of the core layer. For both industrial boards about 3 mm of the material were removed from each side. One-ply boards made of industrial microchips were used as reference for the face layers. The microchips were glued with the same resin at 10% dry weight of the glue per dry weight of the chips. Then, boards with assumed thickness of 3.0 mm, 4.5 mm and 6.0 mm and density of 850 kg/m^3^ and 950 kg/m^3^ were manufactured. Tests were performed to establish a correlation between the modulus of elasticity and bending strength and density. The determined parameters were compared with the properties of the three-ply boards manufactured according to data obtained in earlier studies. The face layer was made of the same type of microchips. The three-ply boards were assessed as described in binding guidelines set out in EN 312 standard [27]. Statistica 13.0 software (StatSoft Inc., Tulsa, OK, USA) was used for statistical analysis. The number of samples analyzed, after outlier elimination, ranged from 9 to 21. The thinner the boards, the more samples were obtained.

**Figure 1:**
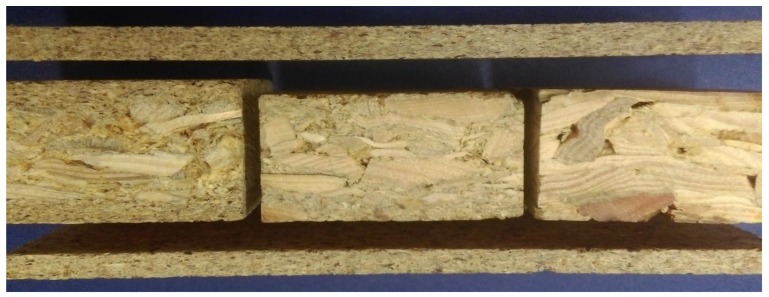
Manufactured boards: below and above-microchips boards, from left to right: chip-sawdust with outer layer from microchips (FRI), chip-sawdust (PT) and chipboards (PZ).

On Figure 1 are presented manufactured boards.

## 3. Results and Discussion

In terms of dimensions and fractional composition the chips were typical for those generated as sawmill waste (Table 1). They contained a large share of a relatively fine fraction (below 18 mm). In this form, they could mainly be used to produce particleboards. Their considerable advantage was a minimal content of bark. The material defined as sawdust contained a fraction of splinter chips of 4 mm or larger, and a finer fraction below 2 mm comprising wood dust, very fine sawdust, and fibrous chips (Table 2). The fraction of 2 and 2.5 mm was a mixture of splinter chips and coarse sawdust, predominated by the latter. In general, the material consisted of 34% of wood chips, 36% of sawdust, and 30% of wood dust. This is an unfavorable composition from a technological point of view. Due to its huge external area, wood dust, and fine sawdust can absorb significant amounts of adhesives. In fact, only wood particles in the form of chips are a desirable component to produce boards. Considering the chips to sawdust ratio and the fact that some of the sawdust had favorable dimensions, the final material used for manufacturing PT boards contained only about 25% of low-quality fractions.

Table 3 presents average modulus of rigidity and modulus of elasticity determined for chip and chip-sawdust boards. It shows that for both types of the experimental boards of density 525 kg/m^3^ and 450 kg/m^3^ these parameters reached relatively low values. Increasing the density above 600 kg/m^3^ resulted in clear growth of the modulus of elasticity but the board strength was still rather low. The module of elasticity for the chip-sawdust boards with density above 525 kg/m^3^ and chip boards with density above 600 kg/m^3^ could meet the requirements of EN 312 standard [27] for P2 boards. The highest modulus of rigidity and modulus of elasticity were noted for the chip-sawdust boards of the assumed density 750 kg/m^3^. In this variant, the first parameter exceeded 10 N/mm^2^, and the second reached almost 2700 N/mm^2^. The assumed increment in density by 75 kg/m^3^ translated into incremental improvement in mechanical properties. Tukey’s test showed significant differences between individual variants for both chip and chip-sawdust boards.

**Figure 2:**
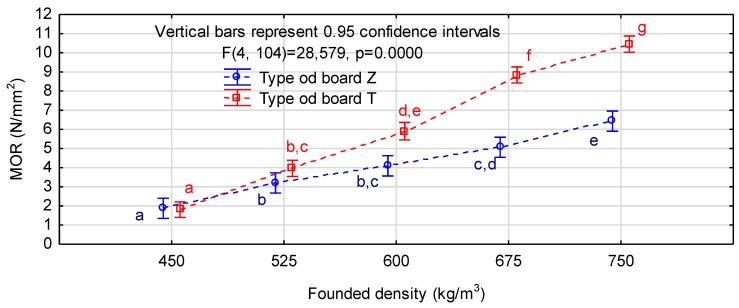
Interaction between density and mat structure and modulus of rigidity of the experimental boards (letters mark homogeneous groups in the HSD Tukey’s test).

**Figure 3:**
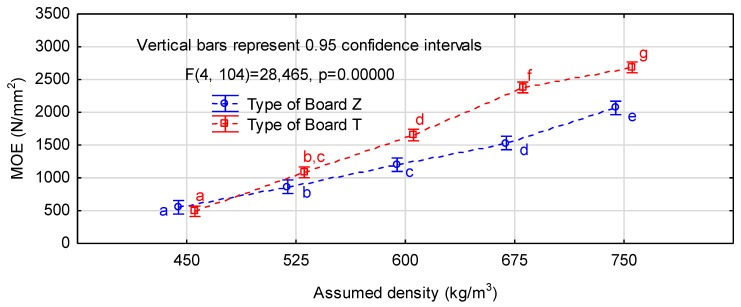
Interaction between density and mat structure and modulus of elasticity of the experimental boards (letters mark homogeneous groups in the HSD Tukey’s test).

Figure 2 and Figure 3 present the results of the interaction analysis for the two factor ANOVA system. They confirm the interaction for both modulus of elasticity and modulus of rigidity, i.e., the positive effects of sawdust presence on the board properties together with increasing the board density. The properties of chip and chip-sawdust boards of densities 450 kg/m^3^ and 525 kg/m^3^ were not significantly different. However, at the density of 600 kg/m^3^, chip-sawdust boards were more advantageous than the chip ones, which reached similar properties only at 675 kg/m^3^. The more homogeneous structure of chip-sawdust boards provided better results at higher densities. In our study, an increase in adhesive consumption by 10% balanced off about 10% savings in wood chip consumption. Sawdust, a low-quality material the management of which is more challenging, improved the board quality, and thus the economic efficiency of the production.

Mechanical properties of the boards largely depend on the board density and the density range is relatively wide. Our study also confirmed strong correlations between the investigated properties and the board density. These correlations are presented in Table 4. The table shows that for both board types lower densities yielded similar values of MOR and MOE. However, as the density increased, greater improvement in MOR and MOE was noted for chip-sawdust than chip boards, as manifested by greater slopes of the curves representing these parameters. Moreover, in the chip boards the MOE to MOR ratio was more stable along with enhancing density. The quotient (a_PT_/a_PZ_) for modulus of rigidity was 2.07, and for modulus of elasticity only 1.57. This relationship is important for modeling three-ply boards with chip or chip-sawdust cores.

Table 5 shows mechanical properties of thick particleboards. As this type of boards is often used for counter tops, the study assessed their both raw and refined variants. The raw boards reached exceptionally high values of the modulus of elasticity. Moreover, MOR 5th percentile for a 38 mm thick board was 8.74 N/mm^2^, and for a 28 mm thick board it was 11.14 N/mm^2^. Therefore, both boards met the requirements set in EN 312 standard [27] for P2 boards. The assessment of the refined board demonstrated its considerable asymmetry with nearly 60% difference in static bending strength. It may be concluded that advantageous properties of this board were due to the refining process, as the raw board would probably not meet the requirements of the standard.

The last two rows of Table 5 contain modulus of rigidity and modulus of elasticity for the core layers from unrefined boards. The strength of the core layers was determined at 6.3 N/mm^2^ and 7.63 N/mm^2^ for 38 mm and 28 mm thick boards, respectively. These values accounted for about 64% of the board strength. Moduli of elasticity in the core layers of these boards were more similar than in the raw boards and reached about 1600 N/mm^2^. These values suggest that chip or chip-sawdust boards may be effectively used for core layers of three-ply boards provided the current quality of the face layers is maintained. One-ply boards of this type would have to have exceptionally high density to achieve the required mechanical properties. However, current trends advocate lowering the board density. Modifying the structure of the core layer while maintaining the quality of face layers is not a new idea but still a relevant one. Sackey et al. [19] discussed the possibilities of modeling the board properties by changing fractional composition of this layer, while Schneider et al. [28] investigated the effects of wood particle geometry on ensuring proper stiffness of the core layers and achieving required density profile. Their results were consistent with the papers published by Mirski or Benthien [29,30,31], who examined the effects of wood particle quality or the share of individual layers. A characteristic feature of three-ply particleboards, and particularly furniture boards, is strong compaction of their face layers comprising much finer fractions. Proper density profile together with the quality of the face layers greatly account for the mechanical properties of particleboards [32] determined in the bend test. In our study, weight ratio of the face layers and the entire board strongly correlated with the board thickness and decreased along with increasing thickness of the final products. The face:core ratio was 25:75 for the 38 mm thick board and 30:70 for the board 28 mm thick. Assuming 35:65 standard ratio for 18 mm thick boards, approximate share of face layer depending on the board thickness may be calculated using the following formula: SFl = −0.5 Tb + 44 (SFl—share of face layer, Tb—board thickness). The change in the share of individual layers is not a continuous but a step change, adapted to a given situation on the production line. The final thickness of the face layers depends not only on their weight share in the board structure but is also determined by compactness and deformability of both layers [29,30]. Elastic properties of the face layers of the industrial boards were determined according to Bodig and Jayne [33], assuming the board is symmetric and contains four lamellas (1):

(1) $$E_{ef}=\frac{1}{J_{x}}\;\overset{3}{\underset{i=1}{\sum }}E_{i}\left[J_{xi}+A_{i}{\left(d_{i}\right)}^{2}\right]$$

The experimental boards were assessed according to EN 310. The resulting properties are presented in Table 6. Data collected therein clearly show that the modulus of elasticity in thin one-ply boards was significantly different. The ANOVA outcome of p=0.01878 was below the assumed significance level = 0.05 (F (3,52) = 3.6278, p = 0.01878). However, a more detailed analysis indicated that the distributions for individual types of boards were similar, as POST HOC tests were inconclusive. The Tukey’s test showed no significant differences for mean modulus of elasticity of the analyzed boards. LSD (least significant differences) Fisher test revealed that the modulus of elasticity more strongly correlated with the board density than their thickness and identified only two homogeneous groups. This was confirmed by Student’s t-test that negatively verified the hypothesis assuming equality of modulus of elasticity in boards 4.5 mm thick, i.e., with density of 859 kg/m^3^ and 929 kg/m^3^ (t = −2.3567, p = 0.0249). The computed densities of face layers in the industrial boards were similar both among each other and in relation to the laboratory boards of the assumed density 850 kg/m^3^. The modulus of elasticity was slightly higher than in the reference laboratory boards. However, the differences were not large and amounted to approximately 7% to 12%.

The following assumptions were made in our search for minimal density of the core layer in chip and chip-sawdust boards: face layers 3 mm thick; E_Tl_ = 3350 N/mm^2^, board thickness 28 mm; modulus of elasticity of the three-ply board 2600 N/mm^2^. Although the required MOE for P2 boards is 1350 N/mm^2^, our earlier studies showed that MOE of 2400–2800 N/mm^2^ is required for these boards to meet the other standard requirements. Results of these calculations are shown in Table 7.

They indicate that to achieve the desired effect, density of the core layer should be about 730 kg/m^3^ and 630 kg/m^3^ for the chip and chip-sawdust core, respectively. The average density of three-ply boards should be about 750 kg/m^3^ for the first case and 680 kg/m^3^ for the second. Assuming a similar relationship between the strength of the three-ply boards and their core layer, we also evaluated modulus of rigidity. However, it turned out that the three-ply boards with the investigated core layer may not meet the standard requirements for MOR. For these reasons, production of this type of boards was abandoned. Their properties are presented in Table 8.

**Figure 4:**
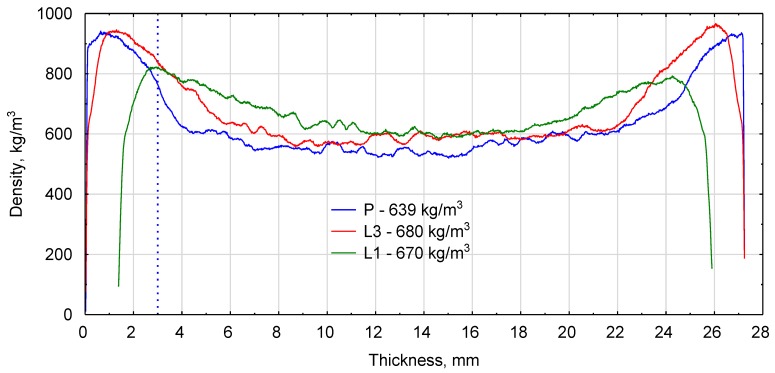
Density profiles: P–industrial board, L1–one-ply chip-sawdust board, L3–three-ply board.

Physical and mechanical properties of the series of model boards presented in Table 8 and Figure 4 indicate that the experimental boards were highly similar to the model ones and met the basic requirements for P2 boards. High compliance with the model was achieved for the face layer thickness and modulus of elasticity. In the industrial boards, 30% of the board weight were located 3.02 mm from the board surface, while in the laboratory boards this distance was 3.12 mm. High density zones were much more clearly outlined in the industrial boards. This was related to the type of press used (continuous-tact), and susceptibility of the core layer to compaction. 

## 4. Conclusions

Unprocessed sawmill by-products in the form of chips and sawdust are valuable materials to produce wood-based boards.

One-ply boards of this type would have to have exceptionally density higher by about 50 kg/m^3^ to 100 kg/m^3^ to achieve the required mechanical properties.

However, they can be successfully used as the core layer of three-layer boards, using the appropriate quality of outer layers.

Much better results may be achieved when the core layer contains a mix of chips and sawdust than chips alone. Sawdust fill empty spaces in the board, making its structure more uniform, so that the board has a better strength.

For the assumed technological parameters, it is possible to produce three-ply boards with properties meeting the criteria for P2 furniture boards.

## Figures and Tables

**Table 1:** Weight and dimensions of the experimental chip.

Mesh Size mm	Weight Share %	Length mm	Width mm	Thickness mm
40	2.32	68.23	32.21	15.21
18	31.72	44.58	26.54	6.49
10	60.76	26.79	11.08	4.20
5	2.89	25.10	5.85	1.67
1	1.84	19.66	2.96	1.39
Mesh fraction	0.47	-	-	-

**Table 2:** Sawdust dimensional analysis.

Fraction (mm)	Share (%)
6.3	0.79 w*
5.0	6.57 w
4.0	12.71 w
2.5	31.93 (9.43 w)
2.0	18.25 (4.51 w)
1.4	25.11
0.315	4.20
Mesh fraction	0.44

* in%, share of wood material that could be considered chips.

**Table 3:** Properties of chip and chip-sawdust boards determined in a bending test.

Type of Board	Chip Boards					Chip-sawdust Boards				
	Density	MOR		MOE		Density	MOR		MOE	
	kg/m^3^	N/mm^2^	%*	N/mm^2^	%	kg/m^3^	N/mm^2^	%	N/mm^2^	%
450	460	1.88^a^	14.6	550^a^	6.8	454	1.80^a^	17.8	491^a^	18.0
525	530	3.20^b^	12.3	886^b^	7.9	547	3.96^b^	18.6	1086^b^	17.8
600	604	4.09^c^	14.1	1201^c^	17.2	608	5.90^c^	12.2	1653^c^	13.0
675	680	5.06^d^	10.0	1533^d^	5.7	696	8.84^d^	13.0	2380^d^	9.3
750	775	6.43^e^	15.3	2068^e^	6.5	761	10.46^e^	12.3	2687^e^	4.2

*—coefficient of variation. a, b, c, d, e—The letters mark uniform groups determined with the HSD Tukey’s test (Tukey’s honest significant difference test), MOR—modulus of rupture, MOE—modulus of elasticity.

**Table 4:** Regression results for MOR/MOE–DENSITY relationship for boards with a linear model (MOR/MOE = b + a).

Board Type	Parameter	Slope, a N/mm^2^·m^3^/kg	b N/mm^2^	r^2^* -	p -	r ** -
PZ	MOR	0.0140	−4.4306	0.9947	0.0000	0.9426
FRI	MOR	0.0290	−11.613	0.9954	0.0000	0.9626
PZ	MOE	4.7600	−1659.1	0.9971	0.0000	0.9756
FRI	MOE	7.4525	−2910.2	0.9926	0.0000	0.9768

*—for mean values-Excel, **—for data set-Statistica.

**Table 5:** Physical and mechanical properties of industrial boards.

Type of Board		Thickness		Density		MOR		MOE	
		mm	%	kg/m^3^	%	N/mm^2^	%	N/mm^2^	%
38	N*	37.92	0.28	602	1.48	9.50	5.21	2408	3.28
28	N	28.20	0.39	637	1.99	12.27	7.68	2803	6.48
28	L	28.73	0.19	634	0.95	9.71	4.98	2767	2.4
28	P	28.73	0.36	640	0.46	15.41	5.64	2734	1.93
38	CL	31.44	1.46	549	1.30	6.30	8.65	1643	2.99
28	CL	21.74	0.95	573	1.14	7.63	5.03	1523	4.22

*—raw, L—pressure on the veneer side, P—pressure on the balance paper side, CL—core layer.

**Table 6:** Physical and mechanical properties of one-ply laboratory boards made from microchips and face layers typical for industrial boards with nominal thickness of 38 mm and 28 mm.

Chip	Thickness	Density		MOE		
	X	x	-	x*	SD	-
	mm	kg/m^3^	%	N/mm^2^	N/mm^2^	%
Lab. 850	3.0	855	4.50	3348.6^a^	410.12	12.25
Lab. 850	4.5	859	6.40	3347.3^a^	349.82	10.45
Lab. 950	4.5	929	3.34	3675.5^b^	380.66	10.35
Lab. 950	6.0	939	4.45	3676.1^b^	314.61	8.56
Computed values						
38 mm	6	859	-	3525	-	-
28 mm	6	859	-	4005	-	-

*—letters mark the results of group homogeneity analysis in the LSD test.

**Table 7:** Calculated parameters of the model board.

E_Cl_	_Cl_ Z	_Cl_ T			MOR CL_Z_	MOR CL_T_	MOR Z	MOR T
N/mm^2^	kg/m^3^	kg/m^3^	kg/m^3^	kg/m^3^	N/mm^2^	N/mm^2^	N/mm^2^	N/mm^2^
1810	728.8	633.4	~755	~ 680	5.77	6.76	9.28	10.87
Data-based assumptions or correlations:								
Outer layer 1	Table 4	Table 4	Table 6	Table 6	Table 4	Table 4	Table 5	Table 5

**Table 8:** Properties of three-ply chip-sawdust board.

Thickness		Density		MOR		MOE		IB	
mm	%	kg/m^3^	%	N/mm^2^	%	N/mm^2^	%	N/mm^2^	%
27.32	0.35	679.39	1.83	12.8	8.33	2625	5.01	0.37	6.38
TS 2H		TS 24H		WA 2H		WA 24H			
%	%	%	%	%	%	%	%	N/mm^2^	%
13.8	5.0	15.9	4.4	65.7	1.8	72.7	1.93		

IB—internal bond.
